# Complex Interactions between Human Myoblasts and the Surrounding 3D Fibrin-Based Matrix

**DOI:** 10.1371/journal.pone.0036173

**Published:** 2012-04-27

**Authors:** Stéphane Chiron, Carole Tomczak, Alain Duperray, Jeanne Lainé, Gisèle Bonne, Alexandra Eder, Arne Hansen, Thomas Eschenhagen, Claude Verdier, Catherine Coirault

**Affiliations:** 1 Inserm, U974, Paris, France; 2 CNRS, UMR7215, Paris, France; 3 UPMC Univ Paris 06 UM76, IFR14, Paris, France; 4 Institut de Myologie, Paris, France; 5 Inserm, U823, Institut Albert Bonniot, Grenoble, France; 6 UPMC Univ Paris 06, Site Pitié-Salpêtrière, Département de Physiologie, Paris, France; 7 AP-HP, Groupe Hospitalier Pitié-Salpêtrière, UF Cardiogénétique et Myogénétique, Service de Biochimie Métabolique, Paris, France; 8 University Medical Center Hamburg-Eppendorf, Hamburg, Germany; 9 CNRS/Université Grenoble 1, LIPhy UMR 5588, Grenoble, France; Kings College London, United Kingdom

## Abstract

Anchorage of muscle cells to the extracellular matrix is crucial for a range of fundamental biological processes including migration, survival and differentiation. Three-dimensional (3D) culture has been proposed to provide a more physiological *in vitro* model of muscle growth and differentiation than routine 2D cultures. However, muscle cell adhesion and cell-matrix interplay of engineered muscle tissue remain to be determined. We have characterized cell-matrix interactions in 3D muscle culture and analyzed their consequences on cell differentiation. Human myoblasts were embedded in a fibrin matrix cast between two posts, cultured until confluence, and then induced to differentiate. Myoblasts in 3D aligned along the longitudinal axis of the gel. They displayed actin stress fibers evenly distributed around the nucleus and a cortical mesh of thin actin filaments. Adhesion sites in 3D were smaller in size than in rigid 2D culture but expression of adhesion site proteins, including α5 integrin and vinculin, was higher in 3D compared with 2D (p<0.05). Myoblasts and myotubes in 3D exhibited thicker and ellipsoid nuclei instead of the thin disk-like shape of the nuclei in 2D (p<0.001). Differentiation kinetics were faster in 3D as demonstrated by higher mRNA concentrations of α-actinin and myosin. More important, the elastic modulus of engineered muscle tissues increased significantly from 3.5±0.8 to 7.4±4.7 kPa during proliferation (p<0.05) and reached 12.2±6.0 kPa during differentiation (p<0.05), thus attesting the increase of matrix stiffness during proliferation and differentiation of the myocytes. In conclusion, we reported modulations of the adhesion complexes, the actin cytoskeleton and nuclear shape in 3D compared with routine 2D muscle culture. These findings point to complex interactions between muscle cells and the surrounding matrix with dynamic regulation of the cell-matrix stiffness.

## Introduction

*In situ*, muscle cells are physiologically immersed in a three dimensional (3D) network that is crucial for a range of fundamental biological processes including migration, survival and contractile performance [1], [2]. The extracellular matrix (ECM) is a complex structure which provides structural and anchoring support to the cells but also contributes to signalling, directing cell fate and function through dynamic cell-matrix interactions. Unfortunately, most of what is known about cell structure and function *in vitro* derives from studies of monolayer cells plated on rigid substrates such as plastic or glass, which are too simple to mimic the native environment.

Three-dimensional (3D) culture of muscle cells has been proposed to provide a more physiological *in vitro* model of muscle growth and differentiation than routine 2D cultures, thus providing an advanced *in vitro* modelling of skeletal muscle [3], [4]. In addition, the creation of skeletal muscle tissue using engineering methods has tremendous potential for the treatment of lost or severely damaged muscles [5]. The biomaterial scaffold plays a key role in most tissue engineering strategies [3], [6]. To guide the organization, growth, and differentiation of cells in tissue engineered constructs, the scaffold should be able to provide a physical support for the cells, and the chemical and biological cues necessary for the formation of a functional tissue [3]. A number of synthetic materials have been developed to provide well controlled and reproducible 3D support of myocyte culture [7], [8], [9]. Alternatively, natural hydrogels present important advantages for engineering functional muscle, primarily because of their higher capacity to provide appropriate adhesion sites for the cells [10]. In particular, fibrin is an attractive matrix for stem cell differentiation and muscle tissue engineering notably because it can interact with integrins and has the capacity to bind specifically many growth factors [11]. A recent study indicates that fibrin gel improves the survival of transplanted myoblasts, probably through cell-matrix-anchorage signalling [12]. Interestingly, fibrin supports the parallel orientation of myotubes under directed mechanical constraints, and thus replicates some crucial aspects of the native skeletal muscle cell patterning [4], [13], [14]. Fibrin-based engineered muscle tissue has been proposed as a useful tool for the rapid identification of new potential treatment for muscle weakness in muscular disorders [15], [16]. However, the mechanical impact of dimensionality offered by 3D scaffolds on seeded myoblasts is largely unknown. How muscle cells interact within the fibrin environment, and how the 3D dimensionality impacts on the shape of the nucleus, the cytoskeleton organization and the cell differentiation remain to be documented.

The goal of the present study was to characterize the interaction between human myocytes and the fibrin-based extracellular environment. We focused on cell anchorage to the fibrin environment and its potential consequences on the cytoskeleton organization, the nucleus and the myocyte differentiation. The matrix stiffness was also analyzed given that there is growing evidence that substrate stiffness is critical for directing myogenic cell fate and tissue homeostasis [17], [18].

We provide evidence that cell anchorage to the fibrin-based 3D environment has profound effects on cell spreading, actin cytoskeleton organization and nuclear shape of human myoblasts. Moreover, our results revealed complex interactions between muscle cells and their surrounding matrix that are of critical importance for pathophysiological applications of 3D muscle culture and for the engineering of a functional skeletal muscle.

## Materials and Methods

### Human skeletal muscle cells

Experiments were performed using primary human muscle cells expanded from the quadriceps muscle of a 12-year-old boy (Myosix, France) in accordance with the French legislation on ethical rules. Cells exhibited a high purity of myoblasts (98.1% CD56+ cells).

### Generation of fibrin-based human engineering muscle tissues

Human engineered muscle tissues were generated as previously described for neonatal rat cardiomyocytes [19]. 6.6 10^5^ human myoblasts were used per fibrin-based engineered muscle tissues (150 µL). Constructs were maintained in 37°C, 5% CO_2_ humidified cell culture incubator in proliferation medium containing Ham's F10 (Gibco) 20% fœtal bovine serum (Gibco), 1% penicillin-streptomycin (Gibco), 10 ng/mL rhFGF (R&D system), 10^−7^ M dexamethasone (Sigma) and 100 µg/mL aprotinin (Sigma), a protease inhibitor that delayed fibrin degradation. When myoblasts were estimated to be 75–85% confluent, constructs were switched to differentiation medium containing high glucose D-MEM (Gibco), 2% horse serum (Biowest), 1% penicillin-streptomycin and 100 µg/mL aprotinin. All media were half changed every two-three days. For comparison, routine bi-dimensional (2D) monolayer human myoblasts were cultured in parallel using standard plastic culture dishes. All other experimental conditions, including proliferation and differentiation media, and medium changes were kept constant between 2D and 3D cultures.

### Histology and immunofluorescence

The distribution of the cells within human engineered muscle tissue was monitored at different time points of the culture. To this end, constructs were fixed with 4% paraformaldehyde (PFA), rinced in PBS, incubated overnight in 30% sucrose 5% DMSO at 4°C, then 4 h at room temperature in 15% sucrose 2.5% DMSO and 50% OCT and then snap frozen in OCT [20]. Sections of 8–10 µm were stained with toluidine blue. The cell morphology in living 3D myoblasts was analysed using green fluorescent calcein that was visualized by confocal fluorescent microscopy, whereas the fibrin matrix was visualized by confocal reflectance microscopy. For other immunostainings, 3D or monolayer cell cultures were fixed with 4% PFA and then rinsed with phosphate buffered saline (PBS)-Glycine 0.1 M. Tissues or cells were permeabilized with 0.05% Triton X100, and blocked with 5% bovine serum albumin (BSA)-IgG free in PBS. Then, they were incubated overnight with primary antibody diluted in 2% BSA. Primary antibodies were: anti- α5 integrin (Millipore, 1/500), anti-vinculin (Sigma, 1/200), anti-α-actinin (Sigma, 1/200), anti-myosin heavy chain (Millipore, 1/100), anti-FAK (Upstate, 1/100), and anti-α-tubulin (Sigma, 1/200). Human engineered muscle tissues or monolayer cells were then incubated with fluorochrome-conjugated secondary antibody. Nuclei were labelled using DAPI. Actin was stained with fluorescent labelled phalloidin (Interchim, 1/200). The preparations were mounted on slides with fluorescent mounting medium (Vectashield, Vector Labs). Secondary antibodies (Invitrogen 1/400) were: Alexa Fluor 488 goat anti-mouse IgG, Alexa Fluor 568 goat anti-rabbit IgG, or Alexa Fluor 488 donkey anti-mouse IgG. Confocal images were captured with a Leica SP2 system (Leica Microsystems, Germany) and analyzed using Image J software. The longest and shortest lengths of nuclei were measured from stacked images and the major to minor length ratio was used as a marker of round or ellipsoid nuclear shape.

### Nucleic acid isolation and quantification of gene expression

RNeasy (Qiagen) was used to prepare total RNA. Proteinase K step was incorporated according to the manufacturer instruction. For reverse transcription and quantitative RT-PCR, Superscript III (Invitrogen) with random primers was used for cDNA generation and SYBR Green PCR Master Mix (Roche) was used according to manufacturer instructions. Experiments were performed on Light Cycler 480 System (Roche). To normalize expression data, we tested multiple internal control genes including RN18S, RPLP0, and H3F3A. Genorm analysis [21] revealed RN18S as the most stable gene during differentiation and was therefore selected as the normalization gene. Final quantification was performed by determining ddCt analysis using the actual PCR efficiency and using RN18S as the housekeeping gene. Primer sequences are listed in Table 1.

**Table 1:** Primer sequences.

Target genes	Primer forward	Primer reverse
MYOG	CAGTGCCATCCAGTACATCG	GCTGTGAGAGCTGCATTCG
ACTN2	AGCGCTTGGAACACCTGGCT	CCGCACCTCTGTCAGCGACG
MYH3	TGGAGCAGGAGGAGTACAAGA	GGATGGAGAAGATGCCCATA
RN18S1	CATTCGAACGTCTGCCCTATC	CCTGCTGCCTTCCTTGGA
H3F3A	GTCTTCAAAAAGGCCAACCA	TCTGATTCGCAAACTTCCCT
RPLP0	CTCCAAGCAGATGCAGCAGA	ATAGCCTTGCGCATCATGGT

### Protein extraction and Western-Blot analysis

Western-blot analysis was performed on total protein extracts, using RIPA buffer. A similar protocol was used for both 2D and 3D cultures, except that 3D had an additional step of lysis using the FastPrep instrument (QBiogen). Protein concentrations were determined using the bicinchoninic acid (BCA) protein assay kit (Pierce) according to the manufacturer's instructions. Proteins were separated by SDS-PAGE, transferred to nitrocellulose membranes and revealed with the following antibodies: anti-vinculin (Sigma, 1/2000), anti- α5 integrin (Millipore, 1/5000), anti-actin (Sigma, 1/500), anti-FAK (Upstate, 1/1000) and anti-lamin A/C (Santa Cruz, 1/2000). Detection was performed using anti-mouse or anti-rabbit horseradish peroxidase labeled antibodies (Jackson ImmunoResearch, 1/20000). The membranes were revealed with ECL chemiluminescent substrate (Millipore). Light emission was detected with a highly sensitive imaging system (G:Box, Syngene). Optical density (OD) was quantified using Image J software. All quantifications were normalized to lamin A expression and expressed in arbitrary units (a.u).

### Electron microscopy

Electron microscopy was performed on fibrin gel constructs fixed in 2% glutaraldehyde, 2% PFA in 0.1 M phosphate buffer (pH 7.4) for 30 min at room temperature. Gels were then dehydrated at 4°C in graded acetone including 2% uranyl acetate in 70° acetone staining step, before Epon resin embedding. Thin (70 nm) sections were stained with uranyl acetate and lead citrate and observed using a Philips CM120 electron microscope (Philips Electronics NV) and photographed with a digital SIS Morada camera. Same procedures were applied for 2D cultured myotubes plated on Thermanox coverslips (Nunc).

### Atomic force microscopy

AFM measurements were carried out using a commercial apparatus (JPK Instruments, equipped with the Petri dish system). The Petri dish (with a coverslip glued on the lower side) was functionalized using a well known protocol adapted to gel adhesion (i.e. treatment using NaOH 0.1 M, 3-aminopropyl-trimethoxysilane–tetramethoxysilane (APTMS) for 10 min, and then glutaraldehyde 0.5% for 30 min). Fibrin gel without cells or engineered muscle tissues were placed onto the functionalized Petri dish, and maintained at 37°C in the culture medium. Measurements were performed after at least a 15 min stabilization period. MLCT Cantilevers from Veeco™ with a pyramid tip were used (half tip angle θ = 18.75° in our case) and set onto the AFM. These were calibrated in advance using the thermal noise method. Then the cantilever was lowered until it came into contact with the gel, as verified using phase contrast microscopy. Indentation and retraction curves were made and the resulting force-indentation (F-δ) curve was obtained and fitted using the Hertz formula [17] adapted to four-sided pyramid F = 0.75 E δ^2^ tanθ/(1-ν^2^) to determine the Young or elastic Modulus (E). In our case, the Poisson coefficient (υ) was taken equal to 0.5. Several locations (at least 4) were chosen and 4 data points were taken each time in a close neighbourhood, therefore N>16 for each time.

### Statistical analyses

All results are expressed as mean ± SD. Sigma Stat was used for the statistical analysis using either t-test or Mann Whitney test depending on the data distribution. Differences between conditions were considered significant at p<0.05.

## Results

### Myoblast spreading and proliferation within the 3D fibrin-based matrix

**Figure 1:**
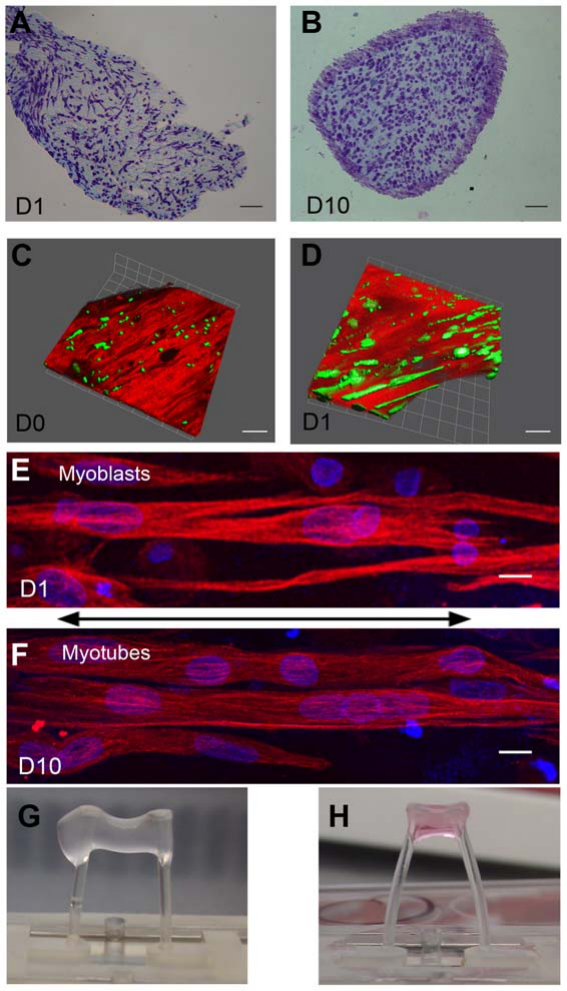
Characterization of the 3D fibrin constructs. A–B: Cell distribution within the fibrin matrix after 1 day (day 1: D1) and 10 days (D10) of 3D culture. Toluidine blue staining of cryostat sections of constructs was analyzed by light microscopy (scale bar = 100 µm). C–D: Cell morphology was analyzed just after gel polymerization (C) (D0) and after 24 hours (D) (D1). Living myoblasts were stained with green fluorescent calcein and visualized by confocal fluorescent microscopy (calcein appeared in green). Fibrin, in red, was visualized by confocal reflectance microscopy. Myoblast alignment was observed as early as day 1. E–F: α-tubulin immunofluorescence visualized by confocal fluorescent microscopy (in red) confirmed the alignment of the myoblasts (E) and myotubes (F) along the gel axis (arrow). Nuclei were stained with DAPI (in blue) (scale bar = 10 µm). G–H: The length of the human engineered muscle tissue greatly reduced overtime due to compaction of the construct. 3D constructs soon after the gel polymerization (G) and 10 days of 3D culture (H). In absence of cells, fibrin gels did not exhibit any compaction.

The distribution of cells within the fibrin-based matrix was uniform at the beginning and all over the time course of the experiment (Fig. 1A–B). Soon after gel polymerization (D0), the myoblasts exhibited a round morphology (Fig. 1C). As early as day 1, myoblasts spread and elongated in the matrix (Fig. 1D) with a predominant longitudinal orientation along the gel axis (Fig. 1D–F). The cell orientation has been attributed to the tension created between the 2 silicone plots [13], [22]. Accordingly, the length of the human engineered muscle tissues greatly diminished with time (Fig. 1G–H).

### Cell adhesion, cytoskeleton organization and nuclear shape

**Figure 2:**
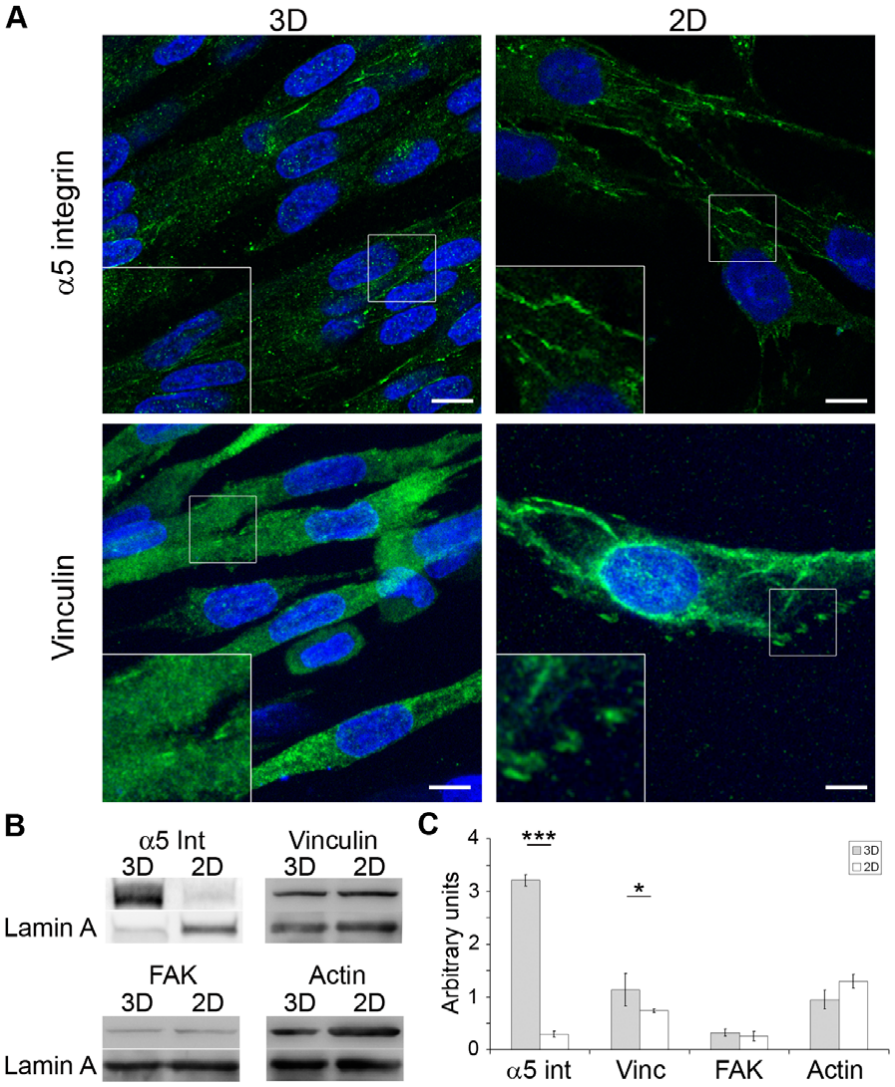
Localization and expression of the adhesion molecules in the 3D constructs. Immunofluorescence staining of myoblasts in 3D fibrin constructs or on routine 2D surface (panel A). Scale bar: 10 µm. Representative Western blots of α5 integrin (α5 int), vinculin (vinc), FAK and actin in 3D (first lanes) and 2D (second lanes) cultures (panel B). Quantification of the Western blot was performed using lamin A as a loading control and expressed in arbitrary units (panel C). Values are means ± SD, n = 4; * p<0.05, *** p<0.001 *vs* value in 2D.

The anchorage of cells into the 3D fibrin-based matrix was investigated after cell spreading by analyzing the organization of the integrin-based cell adhesions (Fig. 2A). After 48h of culture, immunostaining for α5 integrin revealed short adhesion structures that were distributed all over the cell. Similar findings were observed with vinculin, a cytoskeletal protein associated with cell-matrix adhesions (Fig. 2A). In contrast, 2D myoblasts cultured on flat surfaces showed the usual elongated patterns of adhesion molecules whose localization was restricted to the periphery (Fig. 2A) and to the bottom cell-surface interface.

To test whether the morphological differences in cell-substrate adhesions between 3D and 2D cultures were due to differences in protein expression, we evaluated the expression of the adhesion molecules (Fig. 2B). Immunoblot analysis revealed that α5 integrin and vinculin protein expressions were respectively 10 fold and 48% higher in the 3D fibrin matrix compared with 2D cultures (Fig. 2C, each p<0.05). No significant variation in FAK was observed between 3D and 2D.

**Figure 3:**
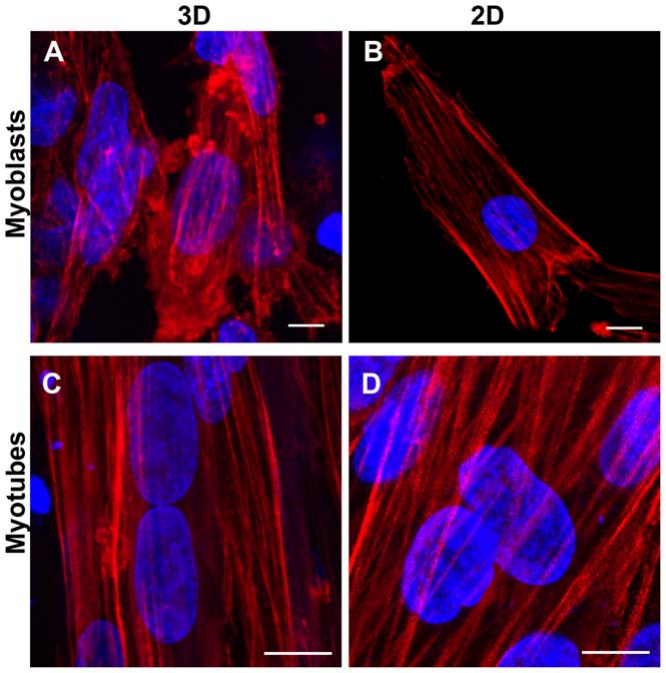
Actin cytoskeleton. Fluorescent phalloidin was used to stain the actin cytoskeleton (in red) in myoblasts (A–B) and myotubes (C–D). Nuclei were stained with DAPI (in blue). Actin stress fibers were observed both in 3D (left panels) and 2D (right panels) cultures but with a different distribution. Note that nuclei in myotubes were aligned in 3D but displayed various orientations relative to actin fiber in 2D. Scale bar: 10 µm.

Because the cytoskeleton network is required for cell spreading, the architecture of the actin cytoskeleton was analyzed in myoblasts embedded in 3D matrix (Fig. 3A). Myoblasts in 3D displayed actin stress fibers that were mainly oriented along the predominant longitudinal direction of gel and cell axis. Actin stress fibers were evenly distributed at the perinuclear regions. In addition, a reticulated network of thinner actin fibers was present at the extremities of myoblasts in 3D. 2D cultured myoblasts were randomly oriented in the culture plate but also displayed actin stress fibers that were mainly oriented along the longitudinal cell axis. However, myoblasts in 2D typically exhibited larger actin fibers at the cell periphery with rare and thinner actin fibers located at the supra-nuclear and subnuclear regions (Fig. 3B). There was no significant difference in the level of actin expression between 3D and 2D myoblasts (Fig. 2C). Interestingly, actin fibers thickened both in 3D and 2D conditions upon myotube differentiation (Fig. 3C–D).

**Figure 4:**
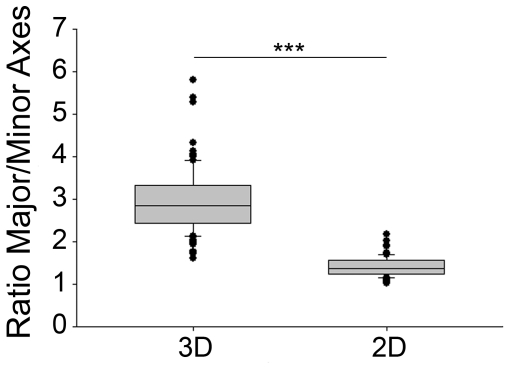
Boxplot analysis of nucleus shape measurements in the 3D fibrin constructs and in 2D conditions. The major to minor length ratio was used as a marker of round or ellipsoid nuclear shape. Values are means ± SD, n = 100 in each. *** p<0.001 *vs* 2D values.

We then examined the nuclear morphology in engineered muscle tissues. Myoblasts in 3D had thick, ellipsoid nucleus that was aligned along the gel axis (Figs. 2, 3 & 4). In contrast, cells on 2D exhibited a round shape and flat nuclei with various orientations with regards to the cell axis. Nuclei were significantly elongated in 3D compared with 2D (each p<0.001) (Fig. 4).

### Myotube differentiation

**Figure 5:**
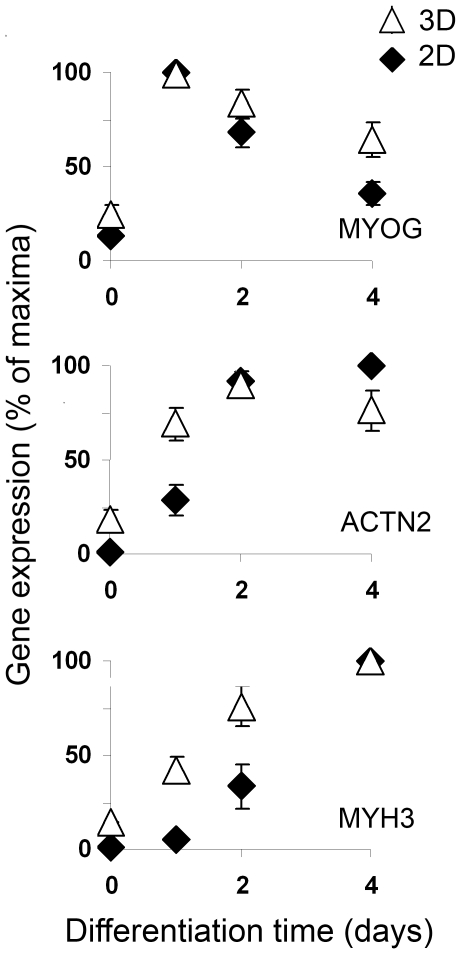
Kinetics of differentiation revealed a faster differentiation in 3D cultures. RT-qPCR of MYOG, ACTN2 and MYH3 during the early time course of differentiation in 3D and 2D cultures. mRNA concentrations of differentiation genes were normalized to 18S expression and expressed as a percentage of maximum. Values are means ±SD, n = 4 independent culture conditions; each qPCR was performed in triplicate, ** p<0.01 *versus* 2D cultures.

Myoblasts proliferated within the fibrin matrix and reached confluence at day 3. After the switch to the differentiation medium, they fused to form long, multinucleated myotubes that were well aligned along the longitudinal axis of the gel (Fig. 1F). This pattern contrasted with the large branched myotubes and random cell orientation usually observed in routine 2D cultures (Fig S1). To evaluate whether the 3D environment modulated differentiation kinetics, early myotube differentiation was monitored by analyzing the transcript levels of differentiation markers (Fig. 5). In both 3D and 2D cultures, MYOG (myogenin) mRNA was essentially absent at the time of media switch to trigger differentiation, reached its maximum after 24 h and then decreased (Fig. 5A). The expression pattern of early differentiation genes including ACTN2 (α-actinin) and MYH3 (myosin heavy chain) mRNAs rose faster in 3D compared with 2D (p = 0.006 and p = 0.004, respectively), and did not drastically differ thereafter. Therefore, the fibrin environment tended to accelerate the pattern of early myotube differentiation.

**Figure 6:**
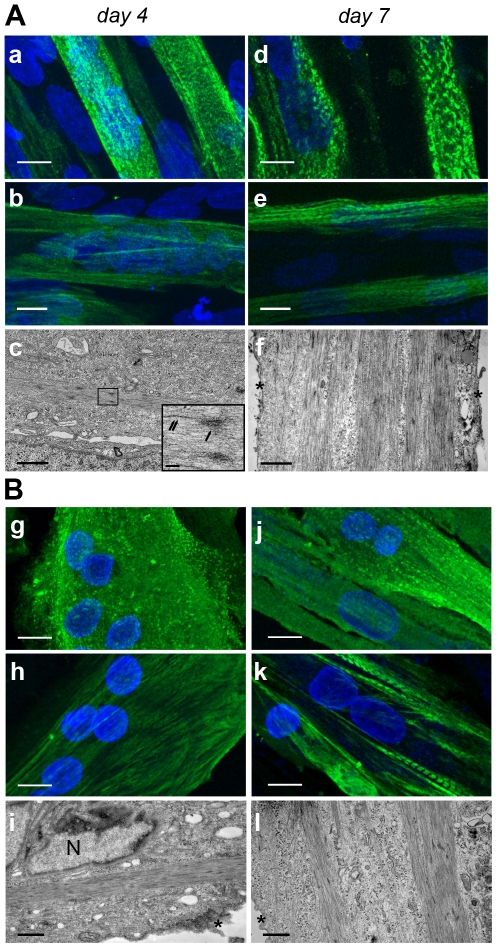
Striation patterns in human engineered muscle tissues (A) and in 2D conditions (B). Immunofluorescence with anti α-actinin (a,d,g,j) and anti-myosin heavy chain (b,e,h,k) 4 and 7 days after the onset of myotube differentiation. Scale bar 10 µm. Panels c,f,i,l: Transmission electron microscopy 4 and 7 days after the onset of myotube differentiation. Single arrow: Z body; Double arrow: thick filament. *: Cytoplasmic membrane; N: nucleus. Scale bar: 1 µm. Insert: close up view of Z bodies; scale bar: 100 nm.

After 4 days of differentiation α-actinin and myosin heavy chain immunostaining of human engineered muscle tissues revealed a partial striation pattern (Fig. 6A, panels a,b) that became more prominent after 7 days of differentiation (Fig. 6A, panels d,e). This was associated with the presence of Z-bodies in the myotubes by day 4 (Fig. 6A, panel c) and their even distribution throughout the entire width of myotubes by day 7 of differentiation (Fig. 6A, panel f). However, there was no marked morphological differences between 3D and 2D as regards to differentiation after 4 and 7 days of differentiation (Fig. 6B).

### Elastic modulus of the fibrin-based scaffold

**Figure 7:**
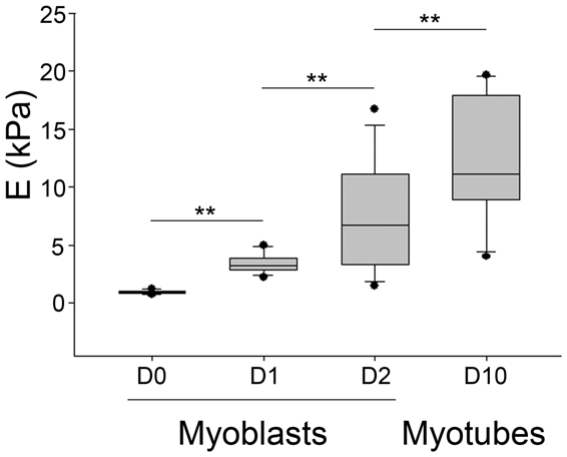
Boxplot analysis of elastic modulus (E) of the human engineered muscle tissues during the course of the culture. E was measured by AFM at the onset of gel polymerization (D0), after 24 h and 48 h of 3D culture in proliferative medium (D1 and D2) and 7 days after the switch from proliferative to differentiation media (D10). At D0, there was no significant difference in E values between gels without cells and gels with cells. E significantly increased during the proliferative period and after myotube differentiation. ** p<0.05.

Because cells sense the elasticity of the surrounding environment and respond by regulating their shape, internal cytoskeleton, and fate in a dynamic pathway, the elastic moduli (E) of the fibrin-based constructs were measured overtime. Soon after gel polymerization, 3D constructs were soft, with E = 1±0.1 kPa (Fig. 7). Spreading and proliferation of the myoblasts were associated with a subsequent increase in the stiffness of human engineered muscle tissues, as attested by significantly higher E values after 24 and 48 h (each p<0.05). The human engineered muscle tissue stiffness further increased upon differentiation, with E reaching 12.2±5.4 kPa after 7 days of differentiation (p<0.05 compared to value at day 2). In absence of cells, the elastic modulus of the fibrin gel did not change over time.

## Discussion

Engineering skeletal muscle tissue fills a critical gap in the currently available physiological tools, between traditional 2D cell cultures and whole animal experiments, with an approach that places cultured cells in an environment that more closely reproduces the complex 3D structure of native tissue. Although the ECM surrounding individual myofibers *in vivo* consists mainly of collagen IV, laminin, and heparin sulphate-containing proteoglycans [23], there is large evidence to suggest that fibrin-based 3D scaffold has great potential for applications in tissue engineering and regenerative medicine [11], [24]. Fibrin networks have been shown to support myocyte anchorage and the formation of the engineered cardiac [19], [25] and skeletal muscle tissue [4], [13], [15], [19]. However characterization of myocyte spreading and adhesions within the 3D environment and cell-scaffold interaction during engineered muscle tissue formation has not been studied before.

In the present study, we show that human myoblasts within a fibrin scaffold spread and form actin stress fibers, even if the low strain modulus of fibrin alone (≃1 kPa) would predict a round cell morphology and the absence of stress fibers [9], [26]. This apparent paradox is consistent with what has been previously reported in mouse NIH 3T3 fibroblasts embedded on fibrin gel [27] and suggests that myoblasts, as fibroblasts or mesenchymal cells, sense and apply strains large enough to enter the strain-stiffening regime of fibrin elasticity [27]. Moreover, our data indicate that the elastic modulus of the fibrin construct increased with the density of myoblasts, thereby indicating that myoblasts also actively stiffened the 3D fibrin. The elastic modulus of the gel further increased upon myotube differentiation (Fig. 7), further supporting the role of inherent cell contractility in the modulation of the fibrin gel stiffness [27]. These findings do not exclude the possibility that factors secreted by myoblasts and/or myotubes into the ECM also modulated the scaffold stiffness during culture [26], a point that deserves further investigation.

Changes in scaffold stiffness have important implications for engineered muscle tissue given that sarcomeres will not form in cultured myocytes unless they grow on a substrate with a stiffness at least equal to physiological muscle stiffness (E approximately 12 kPa) [17], i.e. value higher than that of fibrin alone. Therefore, the capacity of myoblasts to stiffen the fibrin scaffold during the time course of differentiation appears crucial to achieve functional engineered muscle tissue. In addition, it is noteworthy that the final elastic modulus of our construct matched the stiffness of normal skeletal muscle [17], further supporting the formation of a contractile engineered muscle tissue. Myoblast differentiation is a multistep process that involves withdrawal from the cell cycle, acquisition of a cell type-specific transcriptional program and morphological changes that include elongation, alignment and fusion of myoblasts to form myofibers. In both 3D and 2D conditions, the switch from proliferation to differentiation medium was considered as the onset of the differentiation events. Accordingly, the pattern of myogenin expression, an early differentiation marker, did not differ between 2D and 3D. The earlier onset of myosin and α-actinin expression indicated that the 3D environment facilitated myoblast fusion from the early to intermediate stages of differentiation. Reorganization of actin filaments is a known critical factor for myoblast fusion, sarcomere organization, and maintenance of myofibrils [28]. Thickening of actin fibers is part of the actin cytoskeletal remodelling upon myotube differentiation that also includes stress fiber formation, changes in F-actin organization and the shift in the expression of actin protein isoforms from developmental to mature isoforms [29]. Thereby, the elongation and orientation responses of myoblasts along the longitudinal axis of the 3D fibrin gel most probably facilitate end-to-end contact between pre-fusion myoblasts, thereby accelerating the earlier steps of myotubes differentiation compared with 2D (Fig. 5). Because F-actin reorganization is a central event in the induction of cell differentiation, we proposed that in 3D, matrix-induced differences in adhesion proteins and cytoskeletal patterning promoted myoblast alignment, then facilitating fusion of confluent myoblasts and leading to a more rapid differentiation.

The matrix-integrin bonds are the primary links going from the ECM to the inside of the cell [30], [31]. Activation of integrins induces the recruitment of proteins at the focal adhesion sites that in turn facilitates cell adhesion, force transmission and cytoskeletal organization, as well as signalling that modulates cell division, differentiation and apoptosis [32]. We have demonstrated that matrix adhesion sites were present although smaller in size and thus less readily detected in myoblasts embedded in the 3D fibrin gel compared with cells cultured on stiff 2D surface, a finding in agreement with that recently reported for 3D fibroblast cultures [33], [34]. There is evidence that both microenvironment stiffness and dimensionality affect the size and the composition of adhesion sites [33]–[37]. However, the fact that the expression of adhesion proteins was higher in 3D than in 2D cultures could be related to their more uniform distribution at the cell membrane in 3D, thus facilitating cell anchorage to the matrix.

Interestingly, we reported dramatically transformed nuclear morphology in myocytes cultured in 3D (Fig. 4). Thicker and more elongated nuclei observed in our 3D matrix were reminiscent of those physiologically observed in the skeletal muscle tissue (suppl Fig S1) [38]. The precise mechanisms which regulate the shape of the nucleus are not yet fully understood. It has been shown that nuclear shape is at least in part modulated by the interactions between the cytoskeleton and the “linkers of the nucleoskeleton to the cytoskeleton” (LINC) complex that spans the nuclear envelope and in turn anchor networks of filaments to the nucleus [39]. In mouse embryonic fibroblasts cultured on stiff 2D surface, actin has been proposed to form an apical cap to the nucleus that modulates the shape of the nucleus [40]. Disruption of this cap directly or through rupture of the LINC complex increases the thickness of the nucleus [40]. In our cells grown in 3D, actin stress fibers were found around the nucleus, indicating that the presence of an actin cap was not *per se* the unique determinant of the shape of the nucleus. Finally, the presence of actin stress fibers both at the apical and basal sides of the nucleus in human myocytes cultured on 2D may indicate that the perinuclear cap distribution of actin may be cell- and/or species-specific.

In conclusion, our results showed that myoblasts embedded in a fibrin matrix demonstrate mechanotransductive responses by changing the organization of the adhesion complex, the actin cytoskeleton and the shape of the nucleus. This complex myocyte behavior is of critical importance for engineering a functional skeletal muscle tissue and for pathophysiological applications of engineered muscle tissues.

## Supporting Information

Figure S1 **Cell and nuclear morphology of 2D and 3D** ***in vitro*** **myotubes and in muscle tissue.** Immunofluorescence of the human cells with anti-MHC antibody on 2D, 3D myotubes and longitudinal slice of human muscle. Scale bar: 10 µm.
(TIF)
