# Supplementary figures and images for: Complex Interactions between Human Myoblasts and the Surrounding 3D Fibrin-Based Matrix
Source: PLoS One. 2012 Apr 27;7(4):e36173. doi: 10.1371/journal.pone.0036173 (PMC3338613; doi:10.1371/journal.pone.0036173)

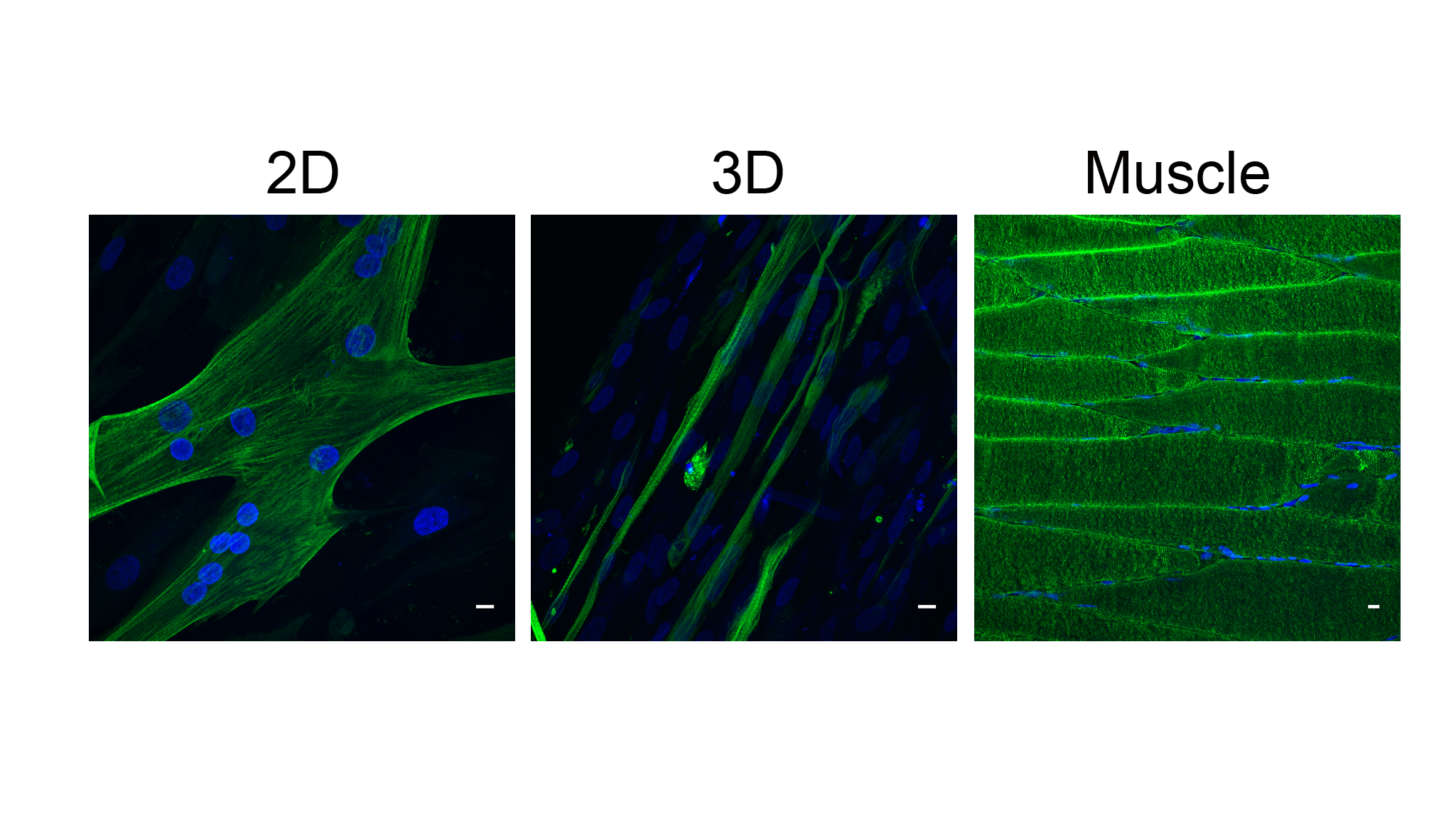

Supplement: Figure S1 — Cell and nuclear morphology of 2D and 3D in vitro myotubes and in muscle tissue. Immunofluorescence of the human cells with anti-MHC antibody on 2D, 3D myotubes and longitudinal slice of human muscle. Scale bar: 10 µm. (TIF) [file pone.0036173.s001.tif]
